# Neuropilin 1 sequestration by neuropathogenic mutant glycyl-tRNA synthetase is permissive to vascular homeostasis

**DOI:** 10.1038/s41598-017-10005-w

**Published:** 2017-08-23

**Authors:** James N. Sleigh, Adriana Gómez-Martín, Na Wei, Ge Bai, Xiang-Lei Yang, Giampietro Schiavo

**Affiliations:** 1 Sobell Department of Motor Neuroscience and Movement Disorders, Institute of Neurology, University College London, London, WC1N 3BG UK; 2 Department of Molecular Medicine, The Scripps Research Institute, La Jolla, California 92037 USA; 3 Discoveries Centre for Regenerative and Precision Medicine, University College London Campus, London, WC1N 3BG UK; 4 UK Dementia Research Institute at UCL, London, WC1E 6BT UK

## Abstract

The mechanism by which dominantly inherited mutations in the housekeeping gene *GARS*, which encodes glycyl-tRNA synthetase (GlyRS), mediate selective peripheral nerve toxicity resulting in Charcot-Marie-Tooth disease type 2D (CMT2D) is still largely unresolved. The transmembrane receptor protein neuropilin 1 (Nrp1) was recently identified as an aberrant extracellular binding partner of mutant GlyRS. Formation of the Nrp1/mutant GlyRS complex antagonises Nrp1 interaction with one of its main natural ligands, vascular endothelial growth factor-A (VEGF-A), contributing to neurodegeneration. However, reduced extracellular binding of VEGF-A to Nrp1 is known to disrupt post-natal blood vessel development and growth. We therefore analysed the vascular system at early and late symptomatic time points in CMT2D mouse muscles, retina, and sciatic nerve, as well as in embryonic hindbrain. Mutant tissues show no difference in blood vessel diameter, density/growth, and branching from embryonic development to three months, spanning the duration over which numerous sensory and neuromuscular phenotypes manifest. Our findings indicate that mutant GlyRS-mediated disruption of Nrp1/VEGF-A signalling is permissive to maturation and maintenance of the vasculature in CMT2D mice.

## Introduction

**Figure 1:**
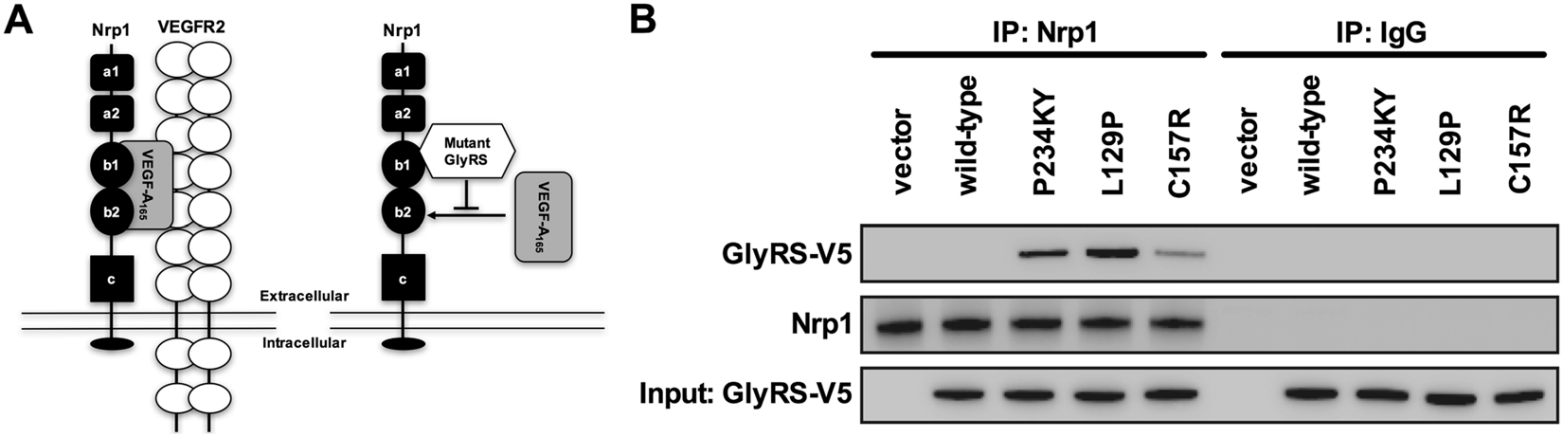
Mutant GlyRS aberrantly binds to the transmembrane receptor neuropilin 1. (**A**) Schematic of Nrp1 and one of its principal co-receptors, VEGFR2, which together bind to the secreted glycoprotein VEGF-A_165_ (on the left)^11^. VEGF-A functions in vasculogenesis, angiogenesis, and arteriogenesis, with the latter two processes occurring through Nrp1 and VEGFR2 signalling^17, 18^. VEGF-A is also critical for nervous system development and maintenance^15^. VEGF-A_165_ binding to Nrp1 is mainly dependent by the b1 domain^12^. CMT2D-associated mutations in *GARS* cause a conformational opening of GlyRS, allowing the aberrant binding of mutant GlyRS to the b1 domain of Nrp1 (on the right)^10^. This competitively antagonises Nrp1/VEGF-A signalling, which contributes to motor deficits observed in CMT2D mice^10^. Schematics are not drawn to scale and adapted from Plein *et al*.^18^ and He *et al*.^10^. (**B**) Co-immunoprecipitation of endogenous Nrp1 showing aberrant interaction with P234KY, L129P, and C157R GlyRS (ectopically expressed with a V5-tag) in NSC-34 cells. Wild-type GlyRS shows no significant binding to Nrp1. The aberrant interaction is weaker with C157R than with P234KY GlyRS, which correlates with the severity of CMT phenotypes in *Gars* ^*C201R*/+^ and *Gars* ^*Nmf249*/+^ mice, respectively.

*GARS* is a widely and constitutively expressed gene that encodes glycyl-tRNA synthase (GlyRS), which serves to covalently attach glycine to its transfer RNA (tRNA), priming it for protein translation^1^. Dominant mutations in *GARS* cause Charcot-Marie-Tooth disease type 2D (CMT2D, OMIM 601472), a currently incurable disorder of the peripheral nerves with the principal clinical symptom of muscle wasting originating in the extremities^2^. A battery of evidence indicates that these *GARS* mutations cause a toxic, gain-of-function in mutant GlyRS^3–8^, and that this is at least partially reliant upon the surface exposure of protein regions usually buried in wild-type GlyRS^9^. Recently published work suggests that these neomorphic regions cause mutant GlyRS to aberrantly bind to the transmembrane receptor neuropilin 1 (Nrp1)^10^, amongst other proteins^8^. Nrp1 functions in both the nervous and vascular systems by modifying multiple signalling pathways^11^. Mutant GlyRS was shown to specifically interact with the extracellular region of Nrp1 integral to VEGF-A_165_ binding (the b1 domain)^12^, and thereby antagonise Nrp1/VEGF-A signalling (Fig. 1A)^10^. CMT2D embryonic day 13.5 (E13.5) hindbrains phenocopy the facial motor neuron migration defects of mice deficient in VEGF-A_164_ (murine equivalent of human VEGF-A_165_) or Nrp1^13^. Moreover, genetically reducing Nrp1 levels enhances the severity of CMT2D pathology, whereas providing additional VEGF-A_165_, via lentiviral intramuscular injections, improves the phenotype^10^.

VEGF-A functions in diverse neuronal processes^14, 15^, and a mild reduction in VEGF-A causes progressive motor neuron degeneration in mice^16^, partially explaining why antagonisation of Nrp1/VEGF-A signalling could be contributing to the neuronal pathology in CMT2D^10^. However, VEGF-A is also critical to blood vessel formation (vasculogenesis), expansion (angiogenesis), and luminal growth of arteries (arteriogenesis), with the latter two processes requiring VEGF-A signalling through Nrp1 and VEGFR2^17, 18^. Nrp1 additionally regulates vascular development by promoting extracellular matrix remodeling downstream of integrins^19^ and by suppressing excessive TGF-β signalling^20^. Although mutating the VEGF-A-binding site of *Nrp1* does not phenocopy the severity of full *Nrp1* knockout mice, it causes a post-natal impairment in angiogenesis and arteriogenesis^21, 22^, resembling the milder phenotype of mice expressing VEGF-A isoforms lacking the Nrp1-binding site^23^. This work suggests that Nrp1 has both VEGF-dependent and VEGF-independent functions in blood vessels, and that Nrp1/VEGF-A signalling is perhaps more important for vascularisation post- than pre-birth^21^. Consistent with this, blocking VEGF-A binding to Nrp1 with a monoclonal antibody impairs vascular remodeling in the peri-natal mouse retina^24^.

Given that 1) mutant GlyRS competes with VEGF-A for Nrp1 binding^10^, 2) GlyRS is secreted^7, 10, 25^ and found circulating in serum of humans and mice^25^, and 3) vascular impairment can contribute to neurodegeneration^26^, we decided to assess the effect of mutant GlyRS on the vascular system of the *Gars* ^*C201R*/+^ murine model of CMT2D.

## Results

### GlyRS^C157R^ aberrantly associates with Nrp1

Six different mutations in *GARS* have been shown to alter protein conformation – five CMT2D-associated mutants^9^ and P234KY corresponding to murine P278KY^10^, which is the spontaneous mutation found in *Gars* ^*Nmf249*/+^, the more severe CMT2D mouse model^4^. Neomorphic Nrp1 binding and disturbance of Nrp1/VEGF-A signalling has been confirmed for a number of GlyRS mutants, including P234KY^10^. In order to verify a common mechanism in the mild *Gars* ^*C201R*/+^ mouse^27^, we performed co-immunoprecipitation assays in NSC-34 cells expressing V5-tagged wild-type, P234KY, L129P, and C157R (human equivalent of C201R) GlyRS. GlyRS^L129P^ was included because it is one of the *GARS* mutants with the best evidence linking it to human neuropathy^28^. As expected, wild-type GlyRS did not bind Nrp1, while GlyRS^C157R^ did, albeit at a lower affinity than the other two mutant proteins (Fig. 1B). The intensity of the aberrant interaction between mutant GlyRS and Nrp1 thus correlates with the overall severity of the mouse model. These experiments confirm that C157R GlyRS spuriously binds to Nrp1 and, extrapolating from findings in the *Gars* ^*Nmf249*/+^ mouse^10^, suggest that the antagonisation of Nrp1/VEGF-A signalling could contribute to the neuropathic phenotype of *Gars* ^*C201R*/+^ mice.

### Nrp1 is highly expressed in blood vessels of skeletal muscles but not motor neurons

**Figure 2:**
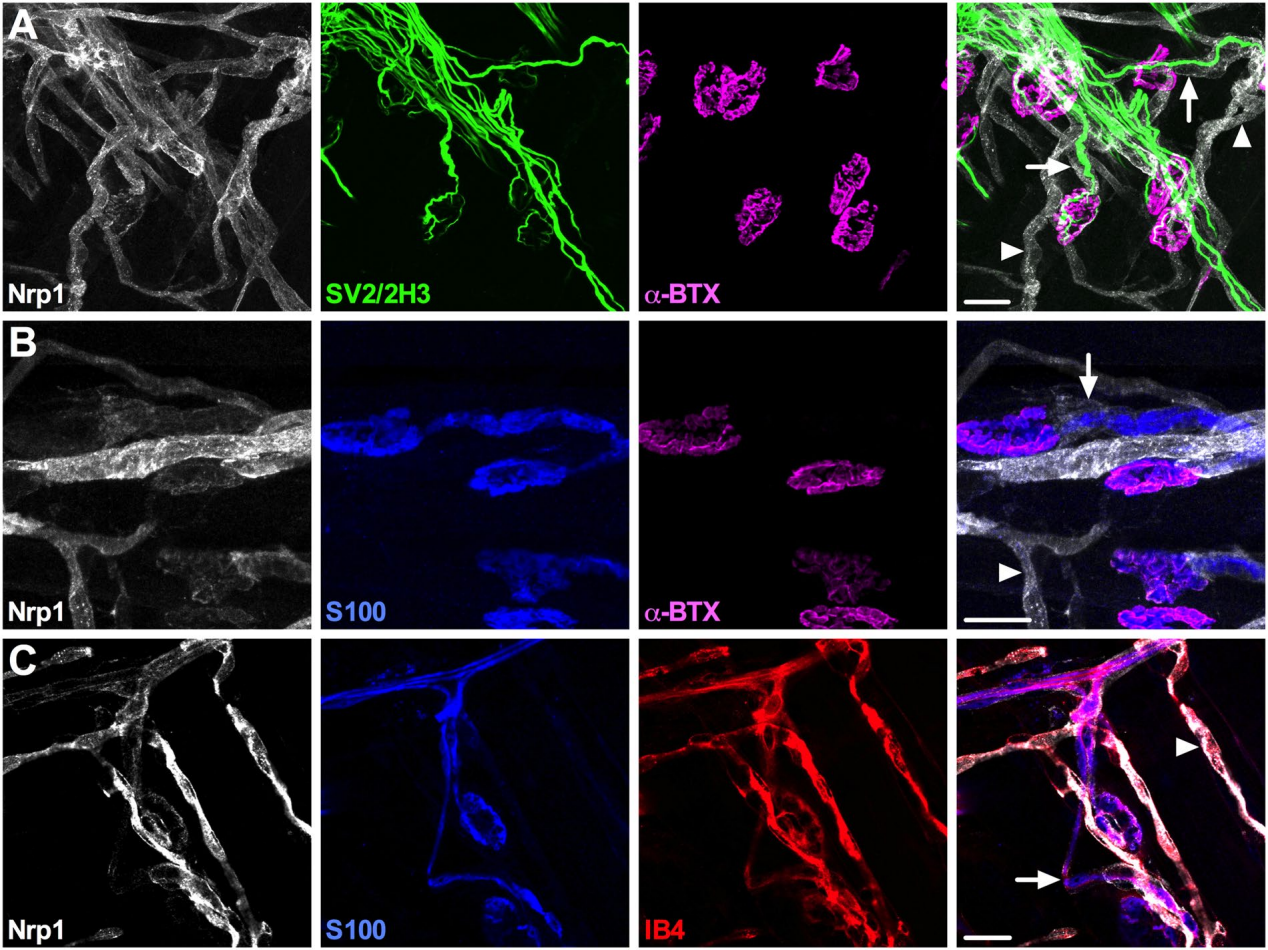
Nrp1 localises to skeletal muscle blood vessels but not motor neurons. Representative Nrp1 staining in one month old wild-type lumbrical muscles. (**A**) Nrp1 (white) localises to structures surrounding, but not perfectly overlapping, lower motor neurons (SV2/2H3, green), and in SV2/2H3 negative areas. α-bungarotoxin (α-BTX, magenta) identifies post-synaptic acetylcholine receptors at the neuromuscular junction. (**B**) Nrp1 staining is not wholly contiguous with the Schwann cell marker S100 (blue) either. This was confirmed with a second glial cell marker, myelin basic protein (Mbp) in Fig. S1C. (**C**) Nrp1 co-localises with IB4 (red), an endothelial cell marker, indicating that Nrp1 is found in muscle blood vessels. Arrows highlight Nrp1 staining associated with motor neurons/myelin, while arrowheads indicate Nrp1^+^ blood vessels. A similar staining pattern was observed at three months and in the TVA muscle at both time points (data not shown). A-B are collapsed Z-stack images and C is a single plane image. Scale bars = 20 μm. See also Fig. S1.

Nrp1 is widely expressed in a range of different tissues^29^, localising to the vascular system^30, 31^ and many neuronal types, including motor neurons^32–34^. As mutated GlyRS binds to Nrp1 impacting the motor nervous system of *Gars* mice^10^, we examined the expression pattern of Nrp1 in wholemount distal, fast-twitch lumbrical and proximal, slow-twitch transversus abdominis (TVA) muscles from one and three month old wild-type mice. These muscles are thin and flat, permitting the visualisation of the entire vascular and nervous systems within the tissue^35, 36^. In these muscles at both time points, Nrp1 (white) did not specifically localise to SV2/2H3^+^ (green) motor neurons or S100^+^ (blue) Schwann cells (Fig. 2A,B). Rather, its expression consistently coincided with the endothelium-binding isolectin B_4_ (IB4, red) (Figs 2C and S1A,B). Nrp1 could be observed faintly surrounding motor neurons, but the staining was wider than that of both S100 (Fig. 2C) and myelin basic protein (Mbp, yellow) (Fig. S1C), suggestive of localisation to blood vessels or closely juxtaposed extracellular matrix encasing the motor neurons and Schwann cells. These results indicate that Nrp1 predominantly localises to the vascular system in post-natal skeletal muscle.

### Blood vessels in Gars^C201R/+^ muscle are unaffected

**Figure 3:**
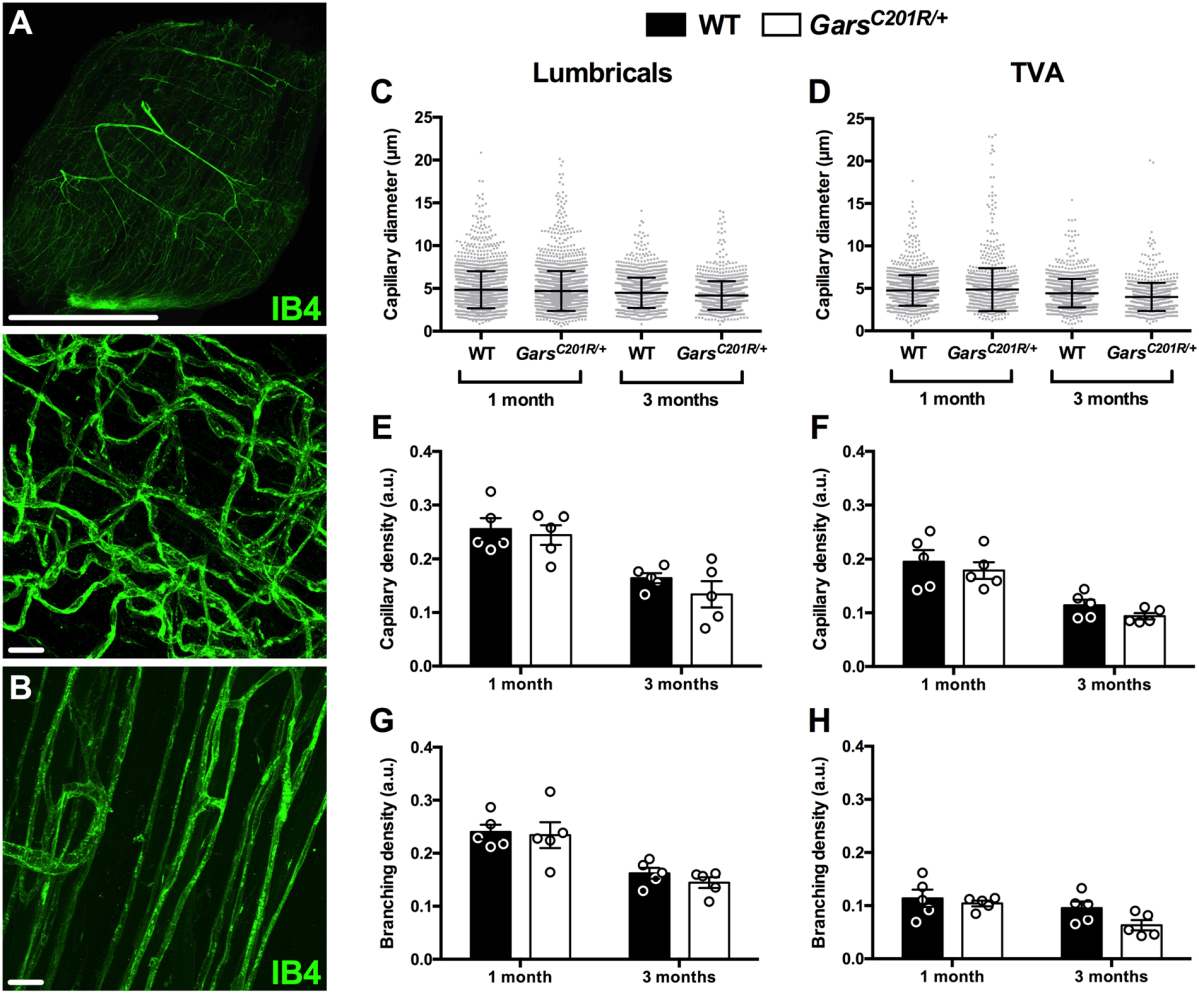
Capillaries appear unaffected in *Gars* ^*C201R*/+^ skeletal muscles. (**A,B**) Representative single plane (A, top) and collapsed Z-stack (**A**, bottom and **B**) images of IB4 (green) staining in one month lumbrical (**A**) and TVA muscles (**B**). Scale bars = 1 mm (**A**, top) and 20 μm (**A**, bottom and **B**). (**C,E,G**) No defects in capillary diameter (C, age, *P* = 0.007; genotype, *P* = 0.090; interaction, *P* = 0.508), density (**E**, age, *P* < 0.001; genotype, *P* = 0.290; interaction, *P* = 0.625), or branching density (**F**, age, *P* < 0.001; genotype, *P* = 0.468; interaction, *P* = 0.721) were observed in *Gars* ^*C201R*/+^ mouse lumbrical muscles at one or three months. (**D,F,H**) The TVA muscle also showed no difference in capillary diameter (**D**, age, *P* = 0.875; genotype, *P* = 0.264; interaction, *P* = 0.359), density (**F**, age, *P* < 0.001; genotype, *P* = 0.223; interaction, *P* = 0.889), or branching density (**H**, age, *P* = 0.020; genotype, *P* = 0.086; interaction, *P* = 0.340). All data sets were analysed with two-way ANOVAs. *n* = 5. See also Fig. S2.

Having shown that C157R mutant GlyRS binds to Nrp1 *in vitro* (Fig. 1B), we assessed structural features of the vascular bed in wild-type and *Gars* ^*C201R*/+^ muscles using IB4 staining (green, Fig. S2). Confocal Z-stack images were taken throughout the entire depth of one and three month old lumbrical and TVA muscles (Fig. 3A,B), and capillary diameter (Fig. 3C,D), density (Fig. 3E,F), and branching (Fig. 3G,H) assessed. These time points represent early and late symptomatic stages of disease in *Gars* ^*C201R*/+^ mice, and were chosen to complement previously performed, in-depth motor and sensory phenotypic analyses^8, 37^. We saw no significant difference between wild-type and mutant *Gars* capillary beds in any of the parameters analysed, suggesting that vasculature is unimpaired in fast and slow twitch skeletal muscles of *Gars* ^*C201R*/+^ mice. The capillary diameter was consistent across muscles and varied little over time, while capillary and branching densities showed variability between muscles, and appeared to decline with age, presumably due to muscle growth. These differences confirm the suitability of this strategy for detecting changes in small blood vessel structure.

**Figure 4:**
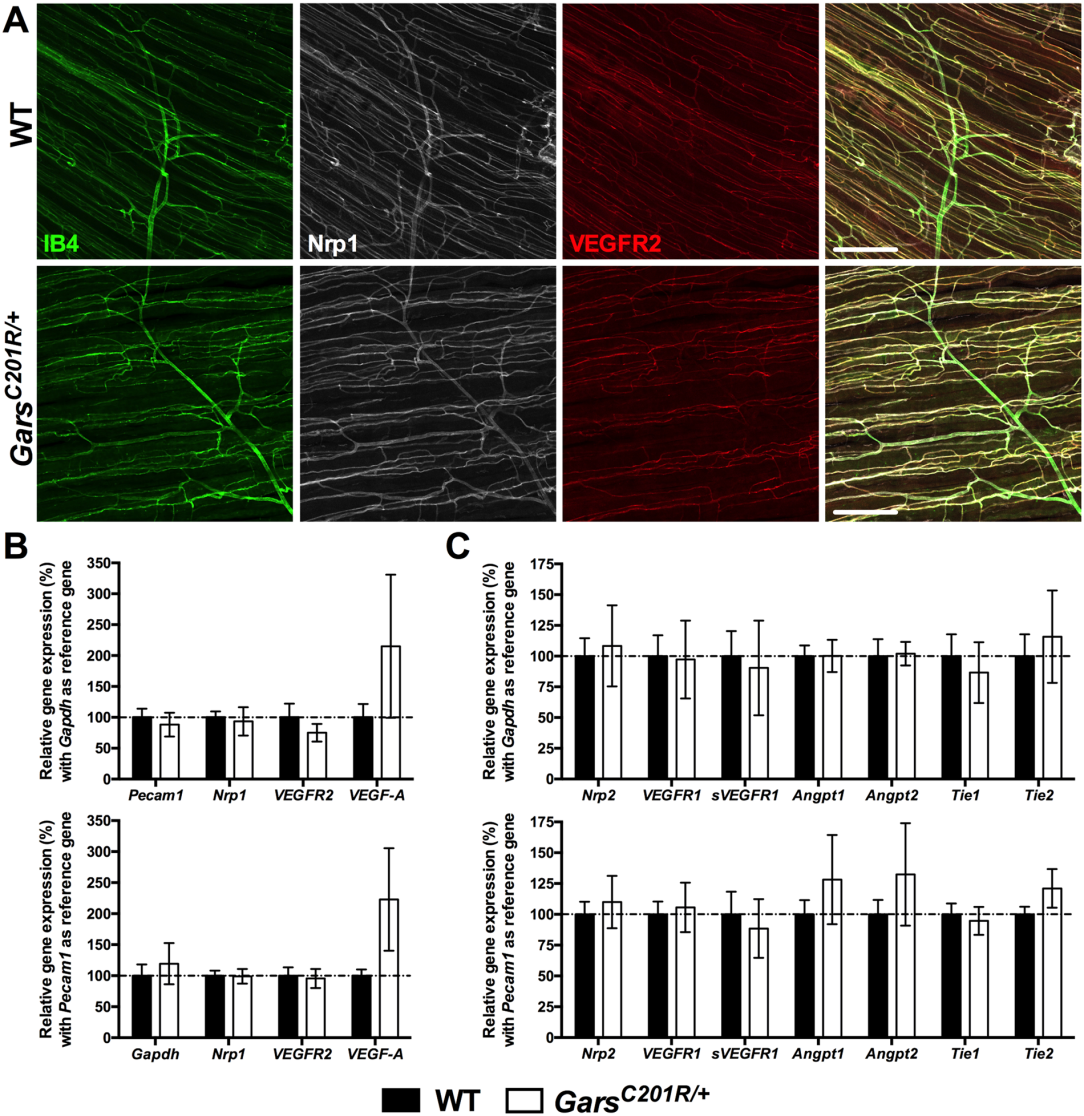
Pathways integral to vascular homeostasis appear unperturbed in mutant *Gars* muscle. (**A**) Representative Nrp1 (white) and VEGFR2 (red) staining in one month old wholemount wild-type and *Gars* ^*C201R*/+^ TVA muscles. Nrp1 and VEGFR2 colocalise with the endothelial marker IB4 (green) in both genotypes, and show no obvious differences. A similar pattern was seen at three months and in the lumbrical muscles at both time points (data not shown). Both panels are single plane images. Scale bars = 200 μm. (**B,C**) qPCR analysis of a series of genes integral to the maintenance of blood vessels indicates very little perturbation in gene expression in one month *Gars* ^*C201R*/+^ tibialis anterior muscles. This was confirmed using either *Gapdh* (top graphs) or endothelium-specific *Pecam1* (bottom graphs) as the reference gene. All individual gene data sets were separately analysed with unpaired, two-tailed *t*-tests. *n* = 6 wild-type and 4 *Gars* ^*C201R*/+^.

Disruption of VEGF-A binding to Nrp1 has previously been linked with reduced Nrp1 expression in various different mouse tissues^21^. We therefore looked at Nrp1 (white) and VEGFR2 (red) protein levels and localisation in lumbrical and TVA muscles, but found no obvious discrepancies between genotypes (Fig. 4A). This was confirmed at the mRNA level in disease-susceptible^27^ tibialis anterior muscles using quantitative RT-PCR (qPCR) with either *glyceraldehyde 3-phosphate dehydrogenase* (*Gapdh*) or endothelium-specific *platelet endothelial cell adhesion molecule 1* (*Pecam1*) as the reference gene (Fig. 4B). Total VEGF-A expression was not significantly increased (Fig. 4B). To rule out the possibility that alternative pathways integral to vascular homeostasis may be contributing to the maintenance of mutant *Gars* blood vessel stability, we also assessed the expression of *Nrp2* ^38^, total and soluble *VEGFR1* (also known as *Flt1* and *sFlt1*, respectively)^14^, and genes integral to angiopoietin-Tie signalling (*Angpt1*, *Angpt2*, *Tie1*, and *Tie2*)^39^. We saw no difference between genotypes in the expression of any of these genes (Fig. 4C), indicating that genetic compensation is unlikely to be playing a crucial role in the maintenance of *Gars* ^*C201R*/+^ vasculature.

### GlyRS^C157R^/Nrp1 binding is permissive to vascular development and homeostasis

**Figure 5:**
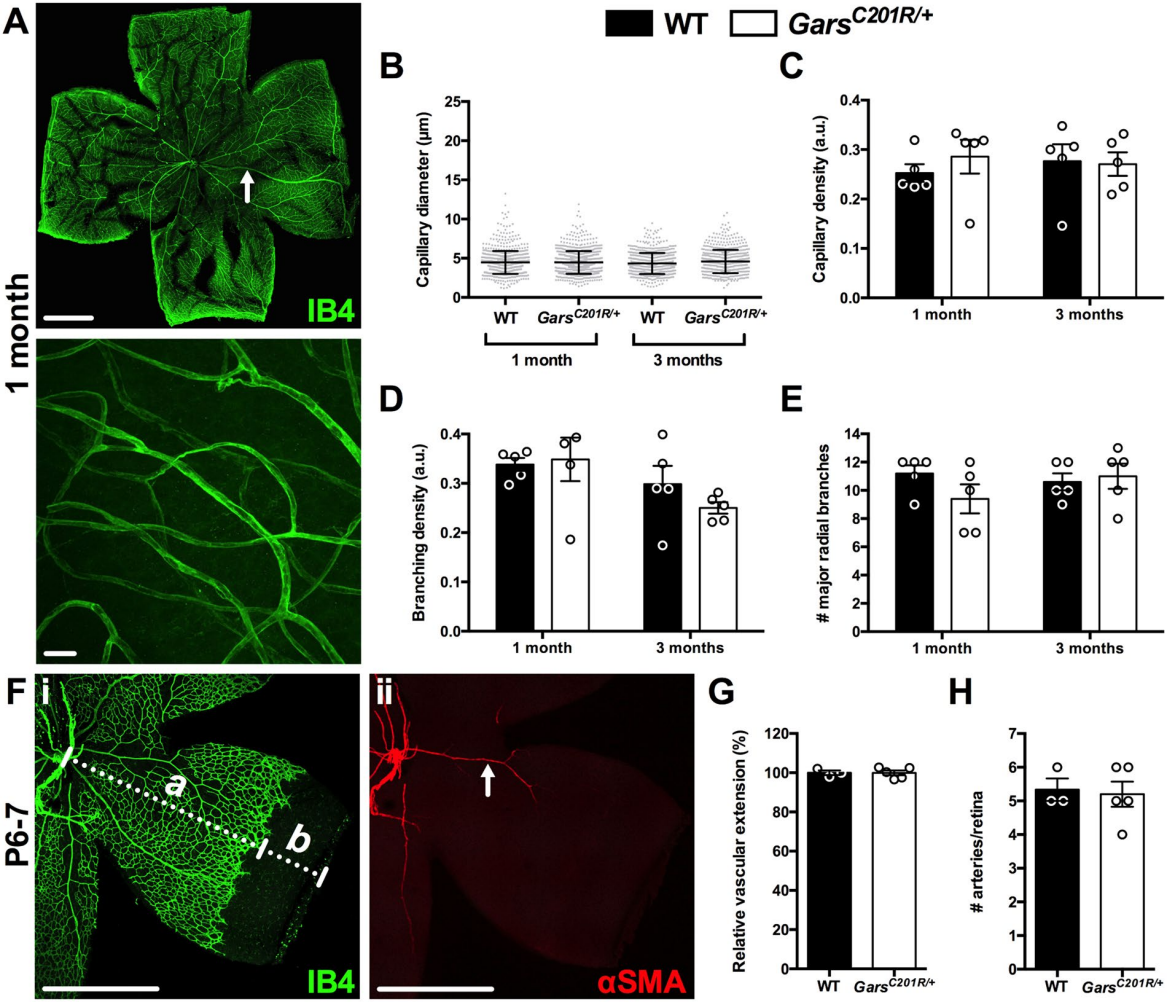
The blood vessel network is unperturbed in *Gars* ^*C201R*/+^ retinas. (**A**) Representative single plane (top) and collapsed Z-stack (bottom) images of IB4 (green) staining in one month retina. Scale bars = 1 mm (top) and 20 μm (bottom). (**B–E**) No defects in capillary diameter (**B**, age, *P* = 0.823; genotype, *P* = 0.112; interaction, *P* = 0.105), density (**C**, age, *P* = 0.878; genotype, *P* = 0.638; interaction, *P* = 0.504), branching density (**D**, age, *P* = 0.042; genotype, *P* = 0.548; interaction, *P* = 0.345), or major radial branch (arteries and veins, arrow in **A**) number (E, age, *P* = 0.541; genotype, *P* = 0.395; interaction, *P* = 0.188) were observed in *Gars* ^*C201R*/+^ mouse retinas at one or three months. *n* = 5. (**F**) Representative single plane images of IB4 (i) and αSMA (red, ii) staining in P6-7 retina. Letters *a* and *b* (i) indicate how vascular extension was assessed (*a* /*a* + *b*) and the arrowhead (ii) highlights an αSMA^+^ artery. Scale bars = 1 mm. (**G,H**) There is no difference in IB4^+^ vascular extension (**G**, *P* = 0.818) or αSMA^+^ artery number (**H**, *P* = 0.967) between wild-type and *Gars* ^*C201R*/+^ P6-7 retinas. *n* = 3 wild-type and 5 *Gars* ^*C201R*/+^. Data sets were analysed with either a two-way ANOVA (**B**–**E**) or an unpaired, two-tailed *t*-test (**G**,**H**). See also Fig. S2.

Nrp1 is indispensable for vascularisation of the mouse central nervous system, but is thought to be less critical, although still important^40^, for the vasculature of other tissues such as muscle^41^. *In vivo* disruption of VEGF-A binding to Nrp1 has previously been shown to disturb blood vessel growth and patterning of the mouse retina^21, 22^. We therefore assessed the IB4^+^ (green) capillary network in one and three month old retinas (Fig. 5A) using an approach similar to that implemented in the muscle analyses (Fig. 3). *Gars* mice had similar retinal capillary diameters (Fig. 5B), densities (Fig. 5C), and branching (Fig. 5D) as wild-type animals at both time points. There was also no difference in the number of major radial branches (arteries and veins) emanating from the central retina (Fig. 5E). Similar to skeletal muscles, no obvious differences in Nrp1 and VEGFR2 staining were seen in mutant *Gars* retinas at either time point (data not shown).

At post-natal day 6–9 (P6-9), vascular extension and artery number are reduced in mice carrying a knockin *Nrp1* mutation that abrogates VEGF-A binding^21, 22, 42^. By adulthood, vascular extension has recovered, but artery number remains diminished^21, 22^. To determine whether mutant GlyRS binding to Nrp1 causes similar nascent phenotypes, we dissected retinas from P6-7 wild-type and *Gars* ^*C201R*/+^ mice and stained them with IB4 (green, Fig. 5F-i) and a fluorophore-conjugated antibody against α smooth muscle actin (αSMA, red, Fig. 5F-ii), an artery-specific marker. Wild-type and mutant *Gars* retinas showed similar vascular extension (Fig. 5G). Furthermore, there was no difference in the number of retinal arteries between genotypes (Fig. 5H), which is consistent with the major radial branch analysis at one month (Fig. 5E).

**Figure 6:**
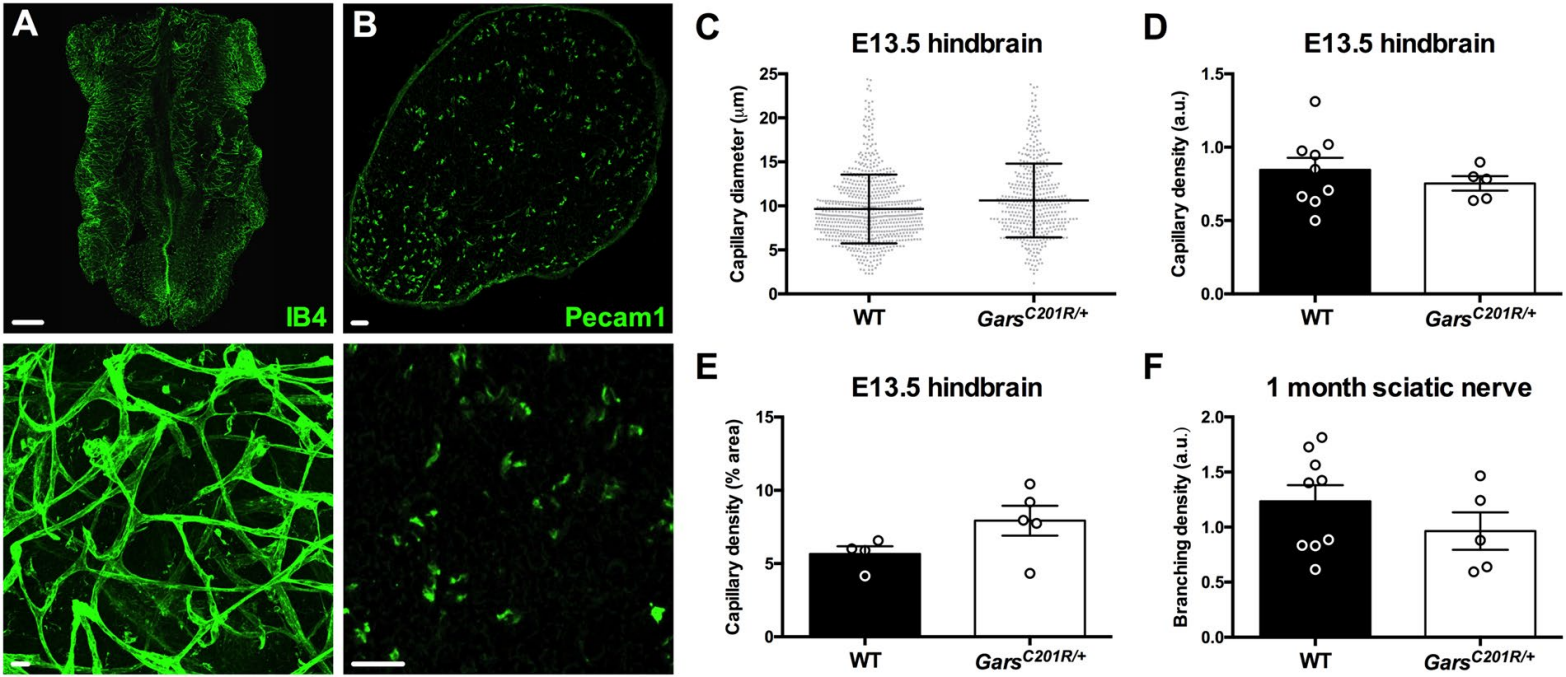
*Gars* mutant embryonic hindbrains and sciatic nerves show no vascular defects. (**A,B**) Representative single plane (**A**, top and **B**) and collapsed Z-stack (**A**, bottom) images of IB4 and Pecam1 staining (green) in E13.5 hindbrain (**A**) and one month sciatic nerve (**B**), respectively. Scale bars = 1 mm (**A**, top) and 20 μm (**A**, bottom, and **B**). (**C–E**) Capillary diameter (**C**, *P* = 0.593), density (**D**, *P* = 0.456), and branching density (**E**, *P* = 0.278) are all unimpaired in *Gars* ^*C201R*/+^ E13.5 hindbrains. *n* = 9 wild-type and 5 *Gars* ^*C201R*/+^. (**F**) Mutant sciatic nerves show no difference in capillary density at one month (*P* = 0.111). *n* = 4 wild-type and 5 *Gars* ^*C201R*/+^. All data were analysed with unpaired, two-tailed *t*-tests. See also Fig. S2.

Fly and mouse models of CMT2D display early developmental defects in the nervous system^7, 8, 10^. We therefore assessed parameters of the capillary network in E13.5 hindbrains of wild-type and *Gars* ^*C201R*/+^ mice. Mutant brains showed no differences in blood vessel structures between genotypes (Fig. 6A,C–E), nor in Nrp1 and VEGFR2 staining (data not shown), which is consistent with western blotting data showing that Nrp1 and VEGFR2 protein levels remain unaltered in embryonic *Gars* neural tissues^10^. This lack of an embryonic vascular phenotype mirrors the result in mice with deficient VEGF-A binding to Nrp1^22^. Finally, we assessed vascular density in sectioned sciatic nerves from one month old mice, in order to determine whether post-natal neuronal tissue was perturbed. Anti-Pecam1 (green) was used instead of IB4 due to superior staining of the vasculature in this tissue (Fig. 6B). Mutant mice showed a non-significant trend towards increased capillary density (Fig. 6F); however, this might simply be caused by the reduced axon calibres (without axon loss) of mutant mice^27^, i.e. the capillaries command a greater percentage area of the sciatic nerve due to the axons being smaller.

## Discussion

Mutant GlyRS appears to compete with VEGF-A for extracellular binding to the transmembrane receptor protein Nrp1 contributing to the peripheral nerve pathology observed in CMT2D^10^. Reduced binding of VEGF-A to Nrp1 has previously been shown to impair post-natal angiogenesis and arteriogenesis^21, 22^. We thus set out to determine whether the capillary network of mutant *Gars* mice is altered either in the post-natal period or during development. First, we confirmed that the human orthologue of murine GlyRS^C201R^ (GlyRS^C157R^), but not wild-type, was capable of aberrantly binding to Nrp1 (Fig. 1B). This interaction is weaker than for GlyRS^P234KY^, which is expressed in *Gars* ^*Nm249*/+^ mice, indicating that mutant disease severity correlates with neomorphic binding affinity. We then found that Nrp1 was highly expressed in post-natal skeletal muscle blood vessels, but not in motor neurons (Fig. 2); in spite of previously published results^43^. This discrepancy may be due low Nrp1 abundance in motor neurons compared to blood vessels and/or differential Nrp1 expression between muscle types (e.g. gastrocnemius versus TVA and lumbricals).

The fundamental requirement for Nrp1 in motor nervous system development is undisputed^32–34, 44^, but its post-natal function and localisation remains less well defined. Motor neuron-specific ablation of Nrp1 using the *Olig2* promoter is reported to cause post-natal motor axon loss and muscle atrophy^45^, but Nrp1 was deleted developmentally^46^ rather than at or post-birth. It is possible that low levels of post-natal Nrp1 expression in motor neurons allows aberrant mutant GlyRS binding and signalling sufficient to drive peripheral nerve degeneration in CMT2D mice. However, given that embryonic defects are observed in *Gars* mice^8, 10^, and that Nrp1 expression is developmentally downregulated in neuronal tissue^47, 48^, we should not rule out that mutant GlyRS-mediated inhibition of Nrp1/VEGF-A signalling during development may be predisposing the motor system to the subsequent degeneration observed at later, post-natal stages^37, 49^.

Given the high levels of Nrp1 expression present in the vasculature of skeletal muscles (Fig. 2), and previously reported in retinas and embryonic hindbrains^30, 31^, we decided to assess capillary architecture in a range of tissues covering the developmental to late symptomatic period. Capillary diameter, density, and branching were unaltered by mutant GlyRS in both distal and proximal skeletal muscles (Fig. 3), as was expression of numerous genes that play a key role in vascular homeostasis (Fig. 4). Lack of a vascular phenotype was replicated in adult retinas (Fig. 5A–E), one month sciatic nerves (Fig. 6F), and embryonic hindbrains (Fig. 6C–E), while P6-9 retinal defects caused by mutating the VEGF-A binding site of *Nrp1* ^21, 22^ were also not present in mutant *Gars* vasculature (Fig. 5F–H). We have therefore shown that the vascular system is unaffected in *Gars* ^*C201R*/+^ mice from embryonic development to adulthood. Extra-neuronal tissue pathology and disease mechanisms have been reported in mouse models of several peripheral nerve conditions including spinal muscular atrophy^50^, Kennedy’s disease^51, 52^, and amyotrophic lateral sclerosis^53^, appearing to mirror the human conditions, at least in some of their most debilitating incarnations^54^. Nevertheless, we have convincingly ruled out that vascular pathology extends to CMT2D, which is in keeping with the clinical presentation of patients.

So why does antagonising Nrp1/VEGF-A signalling affect the peripheral nervous system, but not the vascular system, when this pathway is vital for the functioning of both? First of all, timing and location of Nrp1 and GlyRS expression could conspire to ensure neuronal specificity. GlyRS is secreted and present in serum, but it may not be found in proximity of Nrp1 on blood vessels. For instance, if GlyRS is secreted by terminal Schwann cells at the neuromuscular synapse, mutant GlyRS may only gain access to Nrp1 on motor nerve terminals. Alternatively, the lack of a *Gars* vascular phenotype may reflect that blood vessels require lower levels of Nrp1/VEGF-A signalling than neurons. Finally, mutant GlyRS may be competing with VEGF-A for Nrp1 binding and simply impinging upon VEGF-A-initiated signalling events integral to the nervous, but not vascular, system. Given that VEGF-A is capable of regulating the two vascular-specific processes of angiogenesis and arteriogenesis via distinct pathways^40^, this latter scenario is highly conceivable. Indeed, it is corroborated by the previous observation that VEGF-A-deficient mice display selective degeneration of motor neurons^16^.

In summary, we have demonstrated that CMT2D mice display a pathological phenotype restricted to the nervous system, and that GlyRS-mediated disruption of Nrp1/VEGF-A signalling appears to be permissive to capillary maturation and maintenance. This is consistent with mutant GlyRS binding to Nrp1 affecting a nervous system-specific function of the Nrp1/VEGF-A pathway.

## Materials and Methods

### NSC-34 cell culture and transfection

NSC-34 cell line was purchased from ATCC (Manassas, VA) and grown in Dulbecco’s Modified Eagle Medium (DMEM, Thermo Fisher, Waltham, MA, 41966) supplemented with 10% (v/v) fetal bovine serum (Omega Scientific, Singapore) and 1% (v/v) penicillin-streptomycin (Thermo Fisher, 15140148). Cells were split every two to three days using 0.25% (v/v) trypsin-EDTA (Thermo Fisher, 25200) and cultures maintained at 37 °C in a 5% (v/v) CO_2_ humidified atmosphere. For transfection, NSC-34 cells were grown to ≈70% confluency. Human wild-type, P234KY, L129P, or C157R *GARS* gene was inserted into pcDNA6 plasmid to express wild-type or mutant GlyRS with a V5 tag in NSC-34 cells. Cells were transfected using Lipofectamine 2000 (Invitrogen, Carlsbad, CA, 116680) according to the manufacturer’s instruction.

### Co-immunoprecipitation and western blotting

For cell lysate preparation, 36 h post-transfection NSC-34 cells were washed twice in phosphate-buffered saline (PBS, 137 mM NaCl, 10 mM Na_2_HPO_4_, 2.7 mM KCl, 1.8 mM KH_2_PO_4_), scraped into PBS, pelleted, re-suspended in Pierce IP Lysis Buffer (Thermo Fisher, 87787) for 30 min, and the extract cleared for 7 min at 12,000 × g. Protein G beads (Invitrogen) were pre-incubated with rabbit anti-Nrp1 (Abcam, Cambridge, UK, ab81321) or rabbit IgG (Cell Signaling Technology, Danvers, MA) for 30 min before mixing with cell lysates overnight. The beads were then washed three times with buffer (100 mM NaCl, 50 mM Tris-HCl, pH 7.5, 0.1% (v/v) Triton X-100, 5% (v/v) glycerol), and immunoprecipitates probed by western blot. Immunoprecipitates were fractionated by 4-12% (v/v) Bis-Tris-Plus SDS-PAGE gels (Invitrogen) and transferred to PVDF membranes using the iBlot Dry Blotting System (Invitrogen). Membranes were blocked for 1 h with Tris-buffered saline with 0.1% (v/v) Tween 20 containing 5% (w/v) non-fat dry milk. Wild-type and mutant GlyRS proteins were detected using mouse anti-V5 (1/3000, Invitrogen, R960CUS) and Nrp1 using the same antibody as for co-immunoprecipitation (1/1000). After primary antibody incubation, membranes were washed and probed with HRP-conjugated anti-mouse or anti-rabbit secondary antibodies (Cell Signaling Technology), followed by detection using ECL western blotting substrate (Thermo Fisher) using the FluorChem M imager (ProteinSimple, San Jose, CA).

### Animals

*Gars* ^*C201R*/+^ mice were maintained as heterozygote breeding pairs on a predominantly C57BL/6 background and genotyped as described previously^27^. Mice sacrificed at one and three month time points were 28–34 and 89–97 days old, respectively. Multiple tissues were simultaneously harvested from both males and females. Mouse handling and experiments were performed under license from the UK Home Office in accordance with the Animals (Scientific Procedures) Act (1986), and approved by the University College London (UCL) – Institute of Neurology Ethics Committee.

### Tissue preparation and immunohistochemistry

All steps were performed at room temperature, apart from overnight incubations conducted at 4 °C. Lumbrical and TVA muscles were dissected and immunohistochemically labelled as previously described^35–37^. Eyes were removed and fixed in 4% (w/v) paraformaldehyde (PFA, Electron Microscopy Sciences, Hatfield, PA) for 2 h, before retinas were dissected and stained as reported previously^55^. Sciatic nerves were dissected from mice transcardially perfused with 4% PFA, post-fixed for 2 h, and 10 μm sections processed and stained as previously described^8^. E13.5 hindbrains were dissected and stained using published protocols^56^.

The following primary antibodies were used for immunofluorescence studies: mouse anti-Mbp (1/250, Boehringer, Ingelheim am Rhein, Germany, 1118099), mouse anti-neurofilament (1/50, 2H3, Developmental Studies Hybridoma Bank [DSHB], Iowa City, IA, supernatant), goat anti-Nrp1 (1/100, R & D Systems, Minneapolis, MN, AF566), goat anti-Pecam1 (1/50, Santa Cruz Biotechnology, Dallas, TX, sc-1506), rabbit anti-S100 (1/200, Dako, Glostrup Municipality, Denmark, Z0311), mouse pan anti-synaptic vesicle 2 (1/100, SV2, DSHB, concentrate), and rabbit anti-VEGFR2 (1/100, Cell Signaling Technology, 55B11). When appropriate, tissues were also incubated with 1 mg/ml IB4 biotin conjugate (Sigma Aldrich, St. Louis, MO, L2140) in PBS at 1/50 (retinas) or 1/250 (all other tissues). The following day, combinations of Alexa Fluor-labelled secondary antibodies (Life Technologies, Carlsbad, CA, A-21202, A-21432, A-21447, A-31570, A-31572) at 1/250, 2 mg/ml streptavidin Alexa Fluor 488 conjugate (Life Technologies, S-11223) at 1/250, fluorophore-conjugated α-bungarotoxin (α-BTX, Life Technologies, B-13422 and B-35451; Biotium, Fremont, CA, 00002) at 1/1000, and Cy3-conjugated anti-αSMA (Sigma, C6198) at 1/500 in PBS were used for 2 h, before the tissues were washed and mounted in fluorescent mounting medium (Dako, S3023) for imaging.

### Imaging and analysis

Tissues were imaged using a LSM 780 laser scanning microscope (Zeiss, Oberkochen, Germany) and images analysed using ImageJ software (https://imagej.nih.gov/ij/). All samples were imaged and analysed blinded to genotype. No samples were excluded from the analyses once imaged. IB4-stained muscles, retinas, and hindbrains, and Pecam1-stained sciatic nerve sections were used for capillary analyses as performed previously^54, 57^. Maximal Z-resolution stacked images across the entire depth of wholemount tissues were obtained at 40x (for muscles) and 20x (for retinas and hindbrains) magnification and 1024 × 1024 pixel resolution. Sciatic nerve sections were single plane images taken at 20x and 2812 × 2812 pixel resolution. Three non-overlapping fields of view were imaged per lumbrical muscle, and four lumbricals analysed per animal (12 Z-stacks in total). Eight non-redundant Z-stack images were obtained per TVA and retina, and four per hindbrain, and analysed for each animal. Similar tissue regions were imaged across samples. Five different sections were analysed per sciatic nerve. For measuring capillary diameters, a uniform grid (area per point: 500 μm^2^) was overlaid onto 3D-projected (Max Intensity) images, and every capillary found at a grid intersection measured (except those that were branching), with repeated measures of the same capillary accepted (Fig. S2A,B). Over 400 capillary diameters were measured and averaged per tissue per genotype. For capillary density analyses, images were 3D-projected (Max Intensity), converted to binary (capillaries assigned to black), and particles analysed (Fig. S2A,C). The summed black particle number was then divided by the number of sections to account for Z-stack depth. To assess branching density, capillary bifurcations were counted using the Cell Counter plugin on projected images and the total branches divided by the number of sections (Fig. S2A,D). Non-projected image stacks were used to ensure the counted branches were bifurcations and not crossing capillaries. Vascular extension was assessed in P6-7 retinas by calculating the percentage growth of IB4^+^ vasculature from the centre to the edge of the retina (*a*/*a* + *b*, Fig. 5F-i). To restrict the impact of minor differences in developmental stage between litters, individual extension percentages were normalised to the wild-type mean within the litter.

### RNA extraction and qPCR

Tibialis anterior muscles were dissected from one month old wild-type and *Gars* ^*C201R*/+^ mice, and RNA extracted using an RNeasy Micro Kit (QIAGEN, Hilden, Germany, 74004) as per the manufacturer’s instructions. SuperScript IV (Invitrogen, 18090050) was used as instructed to reverse transcribe 1 μg RNA. Copy DNA was diluted in water (1/10), before qPCR analyses were performed using PowerUp SYBR Green (Applied Biosystems, Foster City, CA, A25742) and a CFX96 Real-Time System (Bio-Rad, Hercules, CA) as previously described^58^. Cycling conditions were as follows: 50 °C for 2 min, 95 °C for 2 min, 40x [95 °C for 3 s, 60 °C for 30 s]. Primers were used at 500 nm and their sequences are detailed in Table 1.

**Table 1:** Primers used for qPCR analysis.

Gene	Ensembl ID	Forward Primer (5′-3′)	Reverse Primer (5′-3′)
*Angpt1*	ENSMUSG00000022309	GCT GAC AGA TGT TGA GAC C	TGT CTG TTG GAG AAG TTG C
*Angpt2*	ENSMUSG00000031465	AGT CCA ACT ACA GGA TTC ACC	TGT CCG AAT CCT TTG TGC
*Gapdh*	ENSMUSG00000057666	TGT GTC CGT CGT GGA TCT GA^58^	CCT GCT TCA CCA CCT TCT TGA^58^
*Nrp1*	ENSMUSG00000025810	GGC TGT GAA GTG GAA GCA CC	AGT GGT GCC TCC TGT GAG C
*Nrp2*	ENSMUSG00000025969	CCG CAC GTT ACT ATT TGA TCC	TCA GAG AAG GTG GAG GAG G
*Pecam1*	ENSMUSG00000020717	ATT GAG GCG CAG GAC CAC G	AAG GAC TCC TGC ACG GTG ACG
*Tie1*	ENSMUSG00000033191	GGG AGA TAG TGA GCC TTG G	CTC GTA CAC TTC GTC ATC G
*Tie2* (*Tek*)	ENSMUSG00000006386	CAC CGA GGC TAT TTG TAC C	CTG TAC TGT TGG CGA TGG
*VEGF-A*	ENSMUSG00000023951	CAT GCC AAG TGG TCC CAG G	CTC AAT CGG ACG GCA GTA GC
*VEGFR1* (*Flt1*)	ENSMUSG00000029648	TAT GCG TGC AGA GCC AGG	GCC ACT GAT GGA GAC CTC G
*sVEGFR1* (s*Flt1*)	ENSMUSG00000029648	TAT GCG TGC AGA GCC AGG	TCC GAG AGA AAA TGG CCT TTT^59^
*VEGFR2* (*Kdr*)	ENSMUSG00000062960	TCT TTG CGC TAG GTA TCC	CTC ACA GAA GAC CAT GCC

*Angpt1*/*2*, angiopoietin 1/2; *Gapdh*, glyceraldehyde 3-phosphate dehydrogenase; *Nrp1*/*2*, neuropilin 1/2; *Pecam1*, platelet endothelial cell adhesion molecule 1; *Tie1*/*2*, tyrosine kinase with immunoglobulin-like and EGF-like domains 1/2; *VEGF-A*, vascular endothelial growth factor A; *VEGFR1*/*2*, vascular endothelial growth factor receptor 1/2; *sVEGFR1*, soluble *VEGFR1*.

### Statistical analysis

Data were assumed to be normally distributed unless evidence to the contrary was provided by the D’Agostino and Pearson omnibus normality test. GraphPad Prism 5 (La Jolla, CA) was used for all analyses. Means ± standard error of the mean are plotted, except for individual capillary diameter data, which represent mean and standard deviation. All analyses were performed on animal mean values.

## Electronic supplementary material

Supplementary Information
