# Supplementary material for: Neuropilin 1 sequestration by neuropathogenic mutant glycyl-tRNA synthetase is permissive to vascular homeostasis
Source: Sci Rep. 2017 Aug 23;7:9216. doi: 10.1038/s41598-017-10005-w (PMC5569042; doi:10.1038/s41598-017-10005-w)
Supplement: Supplementary file 1 — Supplementary Information [file 41598_2017_10005_MOESM1_ESM.pdf]

Neuropilin 1 sequestration by neuropathogenic mutant glycyl-tRNA synthetase is permissive to vascular homeostasis

James N. Sleigh[1,*], Adriana Gómez-Martín[1], Na Wei[2], Ge Bai[2], Xiang-Lei Yang[2], Giampietro Schiavo[1]

[1] Sobell Department of Motor Neuroscience and Movement Disorders, Institute of Neurology, University College London, London WC1N 3BG, UK.
[2] Departments of Chemical Physiology and Cell and Molecular Biology, The Scripps Research Institute, La Jolla, California 92037, USA.

[*] Correspondence to:

James N. Sleigh j.sleigh@ucl.ac.uk Tel: +44(0)20 3448 4334
Fax: +44(0)20 7813 3107

Supplementary Figures

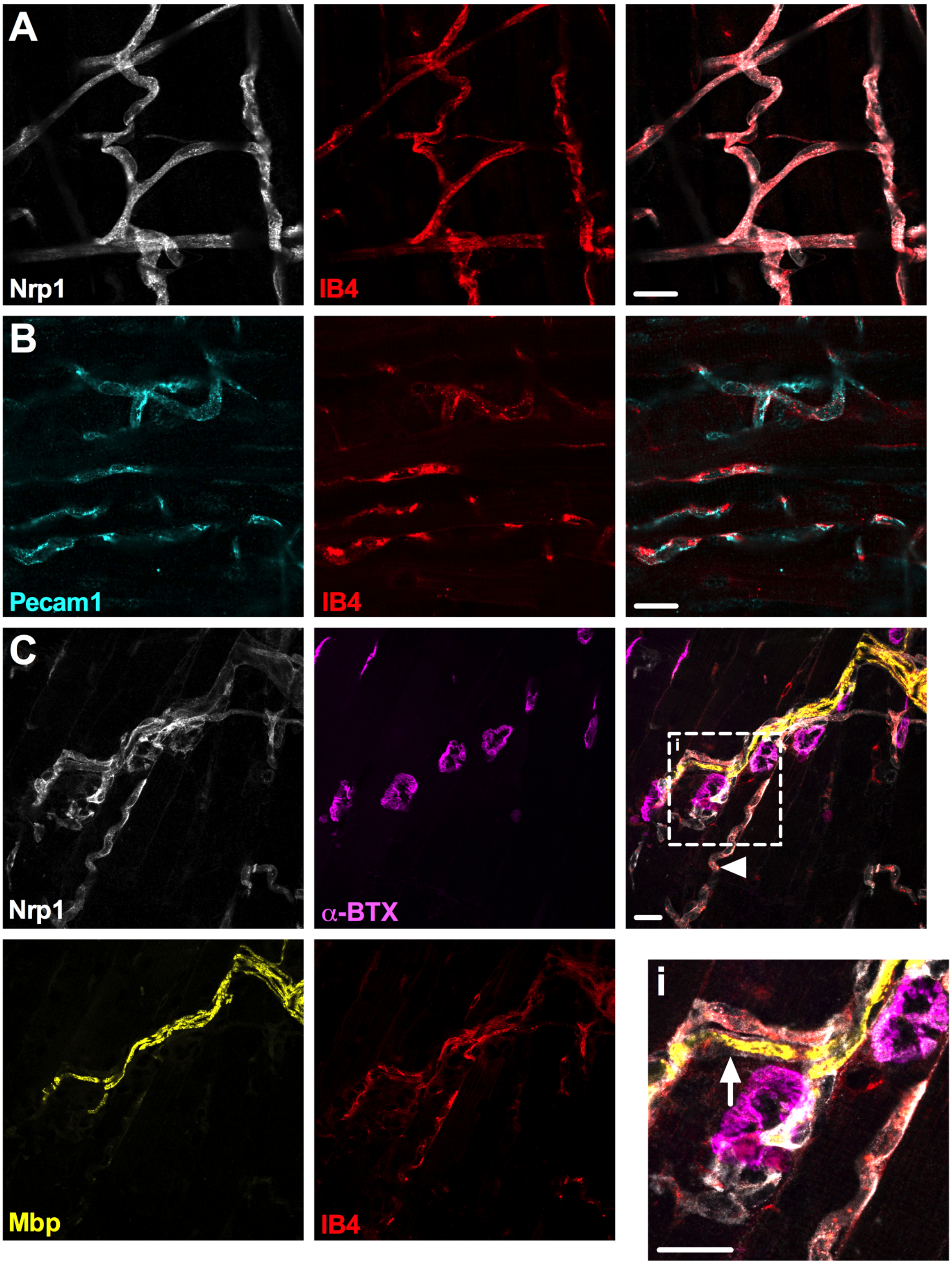

Figure S1. Nrp1 is expressed in IB4$^+$ structures contiguous with the endothelial protein Pecam1. Representative images of Nrp1 localising to capillaries in one month old wholemount lumbrical muscles. (**A**) Nrp1 (white) displays a high degree of co-localisation with IB4 (red), which marks the endothelium. (**B**) IB4 co-localises

with platelet endothelial cell adhesion molecule 1 (Pecam1, cyan), an essential component of the endothelium. (**C**) Nrp1 localises to regions associated with myelin basic protein (Mbp, yellow, arrow), but does not perfectly overlap all glial cells. The arrowhead highlights a $Nrp1^{+}$ capillary. The bottom right panel depicts an enlarged view of the dashed line box in the top right panel. The arrow points to Nrp1 staining surrounding, but wider than, the Mbp^{+} structure, suggesting that Nrp1 is found in blood vessels encasing the motor nerves. Similar staining was observed in the TVA muscle (data not shown). All panels are single plane images. Scale bars = 20 µm.

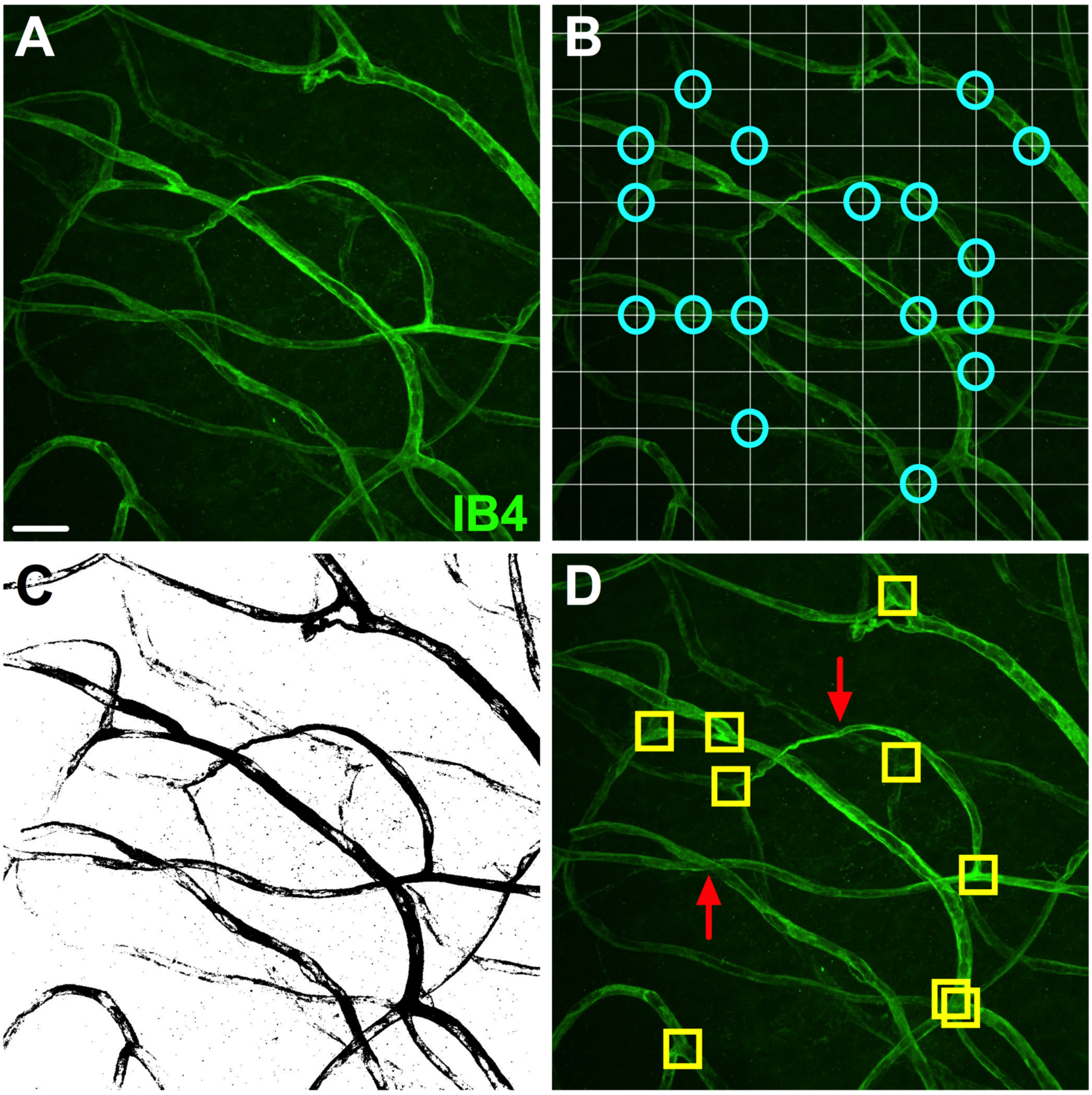

Figure S2. Capillary analyses. (**A**) Representative collapsed Z-stack image of IB4 staining in one month retina. Scale bar = 20 µm, and applies to all images. (**B**) To measure capillary diameters, a uniform grid (white) was projected onto collapsed Z-stack images, and all capillary diameters found at grid intersections measured (cyan circles). (**C**) Collapsed Z-stacks were converted to binary images and the sum of all black pixels calculated using the Analyze Particles tool to measure capillary density. (**D**) Branching density was assessed using the Cell Counter plugin to mark all capillary bifurcations (yellow squares). Non-collapsed stacks were simultaneously used to ensure that crossing capillaries in different planes were not included in the count (red arrow examples).
